# Comprehensibility, reliability, validity, and responsiveness of the Thai version of the Health Assessment Questionnaire in Thai patients with rheumatoid arthritis

**DOI:** 10.1186/ar2796

**Published:** 2009-08-27

**Authors:** Manathip Osiri, Jeerapat Wongchinsri, Sitthichai Ukritchon, Punchong Hanvivadhanakul, Nuntana Kasitanon, Boonjing Siripaitoon

**Affiliations:** 1 Division of Rheumatology, Department of Medicine, Faculty of Medicine, Chulalongkorn University, 1873 Rama IV Road, Pathumwan, Bangkok 10330, Thailand; 2 Department of Medicine, Nopparat Rajathani Hospital, 109 Ramindra Km, 12 Kunnayao, Bangkok 10230, Thailand; 3 Division of Rheumatology, Department of Medicine, Srinakharinwirot University, Princess Maha Chakri Sirindhorn Medical Center, 63 Moo 7, Rangsit-Ongkharak Road, Ongkharak, Nakorn-Nayok 26120, Thailand; 4 Division of Rheumatology, Department of Medicine, Faculty of Medicine, Thammasat University, Paholyotin Road, Amphur Klongluang, Pathumthani 12120, Thailand; 5 Division of Rheumatology, Department of Medicine, Faculty of Medicine, Chiang Mai University, 110 Intawaroros Road, Amphur Muang, Chiang Mai 50200, Thailand; 6 Division of Rheumatology, Department of Medicine, Faculty of Medicine, Prince of Songkla University, 15 Karnjanavanit Road, Hat Yai, Songkhla 90110, Thailand

## Abstract

**Introduction:**

The Health Assessment Questionnaire Disability Index (HAQ-DI) is a commonly used instrument to assess functional status of patients with rheumatoid arthritis (RA). Translations and adaptations of the HAQ-DI have been carried out for use with RA patients in several countries. The objective of this study was to evaluate the psychometric properties of the Thai version of the HAQ-DI (Thai HAQ) in Thai patients with RA.

**Methods:**

Comprehensibility of the Thai HAQ was assessed by 126 patients with RA from 6 medical centers in Thailand. Another group of 115 patients with active RA was enrolled to test the reliability (internal reliability and 1-week test-retest reliability), construct validity (correlations with other measures of RA disease activity), floor and ceiling effects, and sensitivity to change of the Thai HAQ at 3 months of treatment with disease-modifying antirheumatic drugs.

**Results:**

More than 98% of the patients regarded the Thai HAQ as comprehensible. The internal consistency of the Thai HAQ was satisfactory with the overall Cronbach alpha of 0.91. The test-retest reliability of the Thai HAQ was acceptable with the intraclass correlation coefficient of 0.89. Moderate correlations between the Thai HAQ and other outcomes of RA disease activity were observed, except erythrocyte sedimentation rate, with the Spearman correlation coefficients ranging from 0.42 to 0.57. The responsiveness of the Thai HAQ was moderate, with a standardized response mean of 0.75 (95% confidence interval 0.56 to 0.94).

**Conclusions:**

The Thai HAQ is comprehensible, reliable, valid and sensitive to change in the evaluation of functional status of Thai patients with RA. The Thai HAQ is an essential tool to measure treatment effects and progression of disability in RA patients and should be applied in both clinical trials and routine clinical care settings.

## Introduction

The Stanford Health Assessment Questionnaire (HAQ) was originally developed to measure five important health outcomes in patients with chronic diseases [1,2]. These dimensions include premature death, functional disability, pain and discomfort, adverse effects of treatment, and costs. The HAQ Disability Index (HAQ-DI), the HAQ section to evaluate functional capacity, is the most commonly used instrument for assessing disability in patients with rheumatoid arthritis (RA). The HAQ-DI is a predictive factor of future disability and joint damage in patients with RA [3-5]. Because it demonstrated sensitivity to change, the HAQ-DI was chosen by the Outcome Measures in Rheumatology Clinical Trials (OMERACT) and the American College of Rheumatology (ACR) to be incorporated into the core set of outcome measures of RA disease activity [6-8]. The HAQ-DI not only is considered an essential measure of disability in patients with RA in clinical trials, but also is used in clinical practice.

The HAQ-DI has been translated and adapted to suit the activities and cultures in diverse populations from more than 50 countries [9,10]. As with the original HAQ-DI, a number of translations of the HAQ-DI into other languages have been proven to be reliable, valid, and sensitive to change. For the Thai version of the HAQ-DI (Thai HAQ), three items were adapted and two activities were added to the existing items to tailor the questionnaire to the lifestyle and culture of Thai people. The Thai HAQ was back-translated and tested for its validity and responsiveness in a pilot study at a tertiary care hospital in Thailand [11]. However, psychometric validation of the Thai HAQ is still important if it is to be recommended as a standard instrument to measure long-term disability in Thai patients with RA. It is also needed for demonstrating the effectiveness of disease-modifying antirheumatic drug (DMARD) therapy, especially with the expensive biologics, and for use in guidelines to follow the patients over time. Thus, the objective of this study was to evaluate the comprehensiveness, reliability, validity, and responsiveness of the Thai HAQ in Thai-speaking patients with RA from different parts of the country.

## Materials and methods

### Comprehensibility

One hundred twenty-six adult patients who met the ACR 1987 revised criteria for the classification of RA [12] were included in the comprehensibility assessment of the Thai HAQ. These patients were enrolled from six medical centers in Thailand from January to April 2006 regardless of their disease activity. The comprehensibility questionnaire was self-reported by the patients. For older patients and those with poor eyesight, the responses were performed with the assistance of relatives who accompanied them and of rheumatology nurses at the clinics. The patients were asked whether they understood the 20 items from the eight domains of the Thai HAQ. The levels of comprehensibility for each item were categorized on a 4-point scale (0, not comprehensible; 1, slightly comprehensible; 2, moderately comprehensible; and 3, highly comprehensible). Scores of 2 or more for each item were regarded as comprehensible.

### Reliability, validity, and responsiveness

From the same six institutes, 115 more patients with RA were consecutively selected between January 2006 and July 2007 to be included in this part of study. Each patient had to fulfill all of the following criteria: (a) met the ACR 1987 revised criteria for the classification of RA [12], (b) was at least 18 years of age, and (c) had active disease characterized by (i) at least six tender joints, (ii) at least six swollen joints, (iii) a Westergren erythrocyte sedimentation rate (ESR) of at least 28 mm/hour, and (iv) at study entry, just starting a non-biologic DMARD, an increase in the dose of a non-biologic DMARD, or the addition of another non-biologic DMARD. The patients were excluded if they were pregnant or breastfeeding, receiving prednisolone at a dose of more than 10 mg per day, or did not give informed consent. The mode of Thai HAQ administration was similar to that of the comprehensibility assessment. The assessments of reliability, validity, and responsiveness of the Thai HAQ were conducted in accordance with the OMERACT filter for outcome measures in RA [8]. Both parts of the study protocols were approved by the ethics committees of each institute. This study was conducted in accordance with the Declaration of Helsinki. All patients were required to give written informed consent before entering this study.

### The Thai HAQ

The Thai HAQ included 20 items from eight domains adapted from the original HAQ-DI to suit Thai culture and activities. The ability to perform an activity for each item is rated on a 4-level scale, in which the score ranges from 0 (no difficulty in performing that activity) to 3 (inability to perform that activity). The requirement of a device or physical assistance in any item increases the lower score to 2. To calculate the HAQ-DI score, the maximum scores from each domain were summed and divided by 8 to yield a score that ranged from 0 to 3. The higher score indicated greater disability.

### Statistical analysis

Baseline characteristics of the studied patients were presented as number and percentage for discrete parameters and as mean and standard deviation (SD) for continuous parameters. Comprehensibility assessment of the Thai HAQ was presented as percentage of patients with moderate or high comprehensibility in the Thai HAQ.

#### Reliability

Reliability was assessed by test-retest reliability and internal consistency. The test-retest reliability was performed with a 1-week interval. This interval was used because the patients would not be able to remember the first test and the effects of DMARDs added were not expected at 1 week of treatment. The patients completed the first Thai HAQ at their clinic visits. The second was sent to them by mail. Test-retest reliability of the Thai HAQ was analyzed using intraclass correlation coefficients (ICCs). The ICCs and their 95% confidence intervals (CIs) were calculated using a two-way random-effects model. An ICC value of 0.85 or higher was considered acceptable [13]. Internal consistency among each domain of the Thai HAQ was evaluated by Cronbach alpha using the results from the first administration. The overall Cronbach alpha was calculated from all eight domains of the Thai HAQ. For each domain, Cronbach alpha was obtained by deleting that domain from the questionnaire [14].

#### Validity

Construct validity of the Thai HAQ was performed by correlating the baseline eight domains and total scores of the Thai HAQ with the following outcome measures of RA disease activity: number of tender joints (total 68 joints), number of swollen joints (66 joints), patient's assessment of pain, patient global assessment of disease activity, physician global assessment of disease activity, and ESR. Pain score and patient and physician global assessments of disease activity were measured on a 5-level categorical scale, in which the higher score indicated greater pain and worse disease status. The correlation coefficients used in this study were Spearman correlation coefficients. Correlation coefficients of greater than 0.6, of 0.6 to 0.3, and of less than 0.3 were considered strong, moderate, and weak correlations, respectively [15]. The Thai HAQ was also evaluated for floor and ceiling effects. Floor and ceiling effects were considered to be present if at least 15% of the patients scored 0 (the lowest possible score) or 3 (the highest possible score), respectively, on the Thai HAQ [16].

#### Responsiveness

Responsiveness of the Thai HAQ and other measures of RA disease activity was calculated from the baseline values and the values at month 3. To assess the responsiveness of the Thai HAQ and other parameters, the differences between baseline and month 3 scores were used for calculating the standardized response mean (SRM) from the formula:



The SRM value between 0.6 and 0.8 is considered moderate effect and clinically significant. The value of greater than 0.8 represents large effect [17,18]. The statistical software used in this study was SPSS for Windows, version 11.0 (SPSS Inc., Chicago, IL, USA). The statistically significant level was determined as a *P *value of less than 0.05.

## Results

Demographic data of both groups of studied patients are shown in Table 1. The comprehensibility of the Thai HAQ was assessed by 126 patients, whose domiciles were distributed in all parts of the country. Twenty-six patients (20.6%) were from Bangkok. Among the others, 35 (27.8%) resided in Central Thailand, 23 (18.3%) in the North, 11 (8.7%) in the Northeast or East, and 31 (24.6%) in the Southern part of Thailand. The mean (SD) age of the studied patients was 50.5 (13.0) years. One hundred fourteen patients (90.5%) were women. Although 68.3% of the patients (86 patients) completed only primary education (that is, 6 years of formal education), more than 90% of the 126 patients scored each item of the Thai HAQ as moderately or highly comprehensible, as shown in Table 2.

**Table 1:** Demographic characteristics of studied patients

**Demographic characteristics**	**Comprehensibility assessment**	**Reliability, validity, responsiveness assessment**
Number of patients	126	115
Females/males, number (percentage)	114/12 (90.5%/9.5%)	99/16 (86.1%/13.9%)
Age in years, mean ± SD	50.5 ± 13.0	48.9 ± 11.9
Disease duration in months, mean ± SD	ND	68.9 ± 71.8
Rheumatoid factor-positive patients, number (percentage)	ND	88 (76.5%)
Formal education, number (percentage) of patients		
≤6 years	92 (73.0%)	81 (70.4%)
>6 to ≤12 years	26 (20.6%)	24 (20.9%)
>12 years	8 (6.4%)	10 (8.7%)

ND, no data; SD, standard deviation.

**Table 2:** Comprehensibility of each item of the Thai HAQ assessed by 126 Thai patients with rheumatoid arthritis

**Thai HAQ domain**	**Thai HAQ item**	**Percentage comprehensibility**
	Are you able to:	

Dressing and grooming	1. Put on clothes, including buttoning up?	100
	2. Wash your hair?	100

Arising	3. Get up from a chair without armrests?	98.4
	4. Lie down and get up from the bed, or sit in floor-sitting or kneeling position?	100

Eating	5. Slice food with a knife?	99.2
	6. Lift up a glass (filled with water) for drinking?	99.2
	7. Open up food or beverage cans?	99.2

Walking	8. Walk outdoors on level ground?	100
	9. Climb up five steps of stairs?	99.2

Hygiene	10. Apply soap over the body and towel up?	100
	11. Lift up a water bowl to wash yourself?	100
	12. Sit down and get up from a toilet seat?	100

Reach	13. Reach for a 2-kg object from an overhanging cupboard?	98.4
	14. Bend down to pick up an article from the floor?	100

Grip	15. Open a car door?	100
	16. Open containers (such as conserve or Ovaltine jar)?	99.2
	17. Turn on and off a faucet, or wring clothes after washing?	99.2

Activities	18. Go marketing?	99.2
	19. Get on and off a car or a bus?	99.2
	20. Sweep and mop?	98.4

Thai HAQ, Thai version of the Health Assessment Questionnaire Disability Index.

Of the 115 patients with RA enrolled to test the reliability, validity, and responsiveness of the Thai HAQ, 99 (86.1%) were women. The mean (SD) age was 48.9 (11.9) years, and the mean (SD) disease duration was 68.9 (71.8) months. Rheumatoid factor was positive in 77%. The mean (SD) Thai HAQ score at baseline was 1.56 (0.75).

Internal consistency of the Thai HAQ was satisfactory, with the Cronbach alpha of 0.910 among all eight domains. Removal of each domain of the Thai HAQ did not produce a significant change in the Cronbach alpha. The highest alpha was 0.899 when the dressing domain was deleted and the lowest alpha was 0.886 when the reach or activity domain was excluded from the Thai HAQ.

Test-retest reliability of each domain and total Thai HAQ scores was acceptable. The average measure ICC of the Thai HAQ was 0.89 (95% CI 0.84 to 0.92). For each domain of the Thai HAQ, the estimates of ICC ranged from 0.77 to 0.87. The mean and SD of each domain of the Thai HAQ at baseline and day 7 as well as the ICCs and 95% CIs are shown in Table 3.

**Table 3:** Test-retest reliability of eight domains of the Thai HAQ

**Thai HAQ domain**	**Mean ± SD score on day 1**	**Mean ± SD score on day 7**	**ICC (95% CI)^a^**
Dressing and grooming	0.90 ± 0.83	0.82 ± 0.83	0.83 (0.75-0.88)
Arising	2.05 ± 1.67	1.85 ± 0.99	0.84 (0.77-0.89)
Eating	1.60 ± 1.02	1.47 ± 1.02	0.82 (0.75-0.88)
Walking	1.27 ± 0.98	1.12 ± 1.01	0.87 (0.81-0.91)
Hygiene	1.34 ± 0.80	1.22 ± 0.96	0.77 (0.66-0.84)
Reach	1.80 ± 1.07	1.61 ± 1.07	0.77 (0.67-0.84)
Grip	1.91 ± 0.97	1.62 ± 1.00	0.78 (0.69-0.85)
Activities	1.59 ± 1.01	1.33 ± 1.00	0.80 (0.70-0.86)

Thai HAQ	1.56 ± 0.75	1.38 ± 0.80	0.89 (0.84-0.92)

^a^Two-way random-effects average measure intraclass correlation coefficient (ICC). CI, confidence interval; SD, standard deviation; Thai HAQ, Thai version of the Health Assessment Questionnaire Disability Index.

The Spearman correlation coefficients for construct validity of each domain of the Thai HAQ and total Thai HAQ scores are shown in Table 4. Moderate correlation was observed between the majority of Thai HAQ domains as well as the Thai HAQ and outcomes of RA disease activity. These correlation coefficients ranged from 0.30 to 0.57. The highest correlation coefficient was observed between the Thai HAQ and ACR functional class (correlation coefficient 0.57), whereas the lowest was seen between the Thai HAQ and ESR (correlation coefficient 0.37). ESR correlated weakly with four domains of the Thai HAQ (dressing and grooming, walking, grip, and activity domains). The grip and walking domains of the Thai HAQ also had weak correlations with the number of swollen joints, number of tender joints, and pain level.

**Table 4:** Spearman correlation coefficients between each domain and the Thai HAQ and outcomes of rheumatoid arthritis disease activity

**Thai HAQ domain**	**Tender joint count**	**Swollen joint count**	**Patient global assessment**	**Physician global assessment**	**Pain**	**ESR**	**ACR functional class**
Dressing	0.31	0.32	0.40	0.34	0.34	0.23^a^	0.47
Arising	0.42	0.39	0.45	0.42	0.35	0.43	0.35
Eating	0.32	0.33	0.35	0.30	0.31	0.35	0.44
Walking	0.37	0.27^a^	0.43	0.39	0.35	0.28^a^	0.53
Hygiene	0.39	0.39	0.44	0.37	0.33	0.40	0.42
Reach	0.39	0.39	0.39	0.35	0.32	0.31	0.42
Grip	0.29^a^	0.29^a^	0.35	0.33	0.22^a^	0.28^a^	0.38
Activity	0.30	0.35	0.44	0.42	0.41	0.23^a^	0.51

Thai HAQ	0.46	0.43	0.52	0.48	0.42	0.37	0.57

^a^Correlation coefficient of less than 0.3 represents weak correlation and 0.3 to 0.6 represents moderate correlation between the Thai HAQ (Thai version of the Health Assessment Questionnaire Disability Index) domain and rheumatoid arthritis disease activity. *P *< 0.05 for all correlation coefficients. ACR, American College of Rheumatology; ESR, erythrocyte sedimentation rate.

Floor and ceiling effects of the Thai HAQ were not observed in the studied patients. At baseline, none (0%) scored 0 or 3 on the Thai HAQ. At 3 months of treatment, 6 patients (5.2%) scored 0 and 1 patient (0.9%) scored 3.

Responsiveness of the Thai HAQ and other measures of RA disease activity was moderate as the SRMs were 0.6 or higher, except for ESR. The SRM for the patient global assessment of disease activity was the highest (0.94), whereas that for the Thai HAQ was 0.75. The SRMs for the Thai HAQ compared with the other measures of RA disease activity are shown in Table 5.

**Table 5:** Differences of treatment effect and standardized response means of rheumatoid arthritis disease activity and function outcomes between baseline and month 3 of treatment

**Rheumatoid arthritis outcome**	**Mean difference of treatment (95% CI)**	**SRM (95% CI)**
Tender joint count	-3.58 (-2.42 to -4.74)	0.59 (0.40 to 0.78)
Swollen joint count	-2.72 (-1.90 to -3.53)	0.64 (0.45 to 0.83)
Patient global assessment	-0.79 (-0.63 to -0.96)	0.94 (0.74 to 1.13)
Physician global assessment	-0.77 (-0.61 to -0.94)	0.89 (0.70 to 1.09)
Pain level	-0.75 (-0.58 to -0.93)	0.82 (0.62 to 1.01)
ESR	-13.93 (-7.01 to -20.84)	0.41 (0.21 to 0.61)
Thai HAQ	-0.50 (-0.38 to -0.63)	0.75 (0.56 to 0.94)
ACR functional class	-0.44 (-0.29 to -0.60)	0.59 (0.38 to 0.80)

ACR, American College of Rheumatology; CI, confidence interval; ESR, erythrocyte sedimentation rate; SRM, standardized response mean; Thai HAQ, Thai version of the Health Assessment Questionnaire Disability Index.

## Discussion

Approximately 90% of the patients enrolled in this study were women. The high proportion of female patients with RA was observed across all six institutes (range 73.3% to 96.8%). This finding was because more women than men are affected by RA and because women with chronic rheumatic diseases in Thailand generally comply with long-term treatment better than men do.

Our study has shown that the Thai HAQ is comprehensible among Thai patients with RA recruited from different parts of Thailand. Despite the finding that more than 70% of the patients had a limited educational level, there was no significant variation in the comprehensibility of each item of the Thai HAQ. All 126 patients regarded nine items as comprehensible. Only one and two patients, respectively, rated eight and three items of the Thai HAQ as slightly comprehensible. Among the three items with the least comprehensibility, two of these ('get up from a chair without armrests' and 'reach a 2-kg object from an overhanging cupboard') are common activities in many urbanized populations. However, neither of these activities is considered common by older Thai people who get used to living in the traditional Thai style. The other item, sweep and mop, a common activity for Thai people, was rated as slightly comprehensible by two older patients with RA who no longer performed this activity. The comprehensibility of the Thai HAQ was higher than that of the Korean HAQ, which ranged from 76% to 98% [19].

The Thai HAQ scores that included and excluded the use of aids/devices or assistance varied between 0 and 0.25 (data not shown). Use of aids/devices or other people's assistance may enhance the HAQ-DI scores [20]. In this study, most patients who required aids/devices or assistance scored that item 3, instead of 2, as they were unable to do that activity by themselves.

The Thai HAQ has been demonstrated to have satisfactory internal consistency with the Cronbach alpha of 0.910. The Cronbach alpha for each item of the Thai HAQ was also high and varied insignificantly. Our findings were comparable to the results from the HAQ-DI in other Asian countries, including South Korea [19], China [21], Kuwait [22], and Japan [23]. The Cronbach alpha in these studies ranged from 0.86 to 0.95. Test-retest reliability of the Thai HAQ has been shown to be acceptable with the ICC of 0.89. The test-retest reliability of the Thai HAQ was less than those of the Korean and Japanese HAQs but was higher than the Chinese and Arabic HAQs. For each domain of the Thai HAQ, only the walking domain had an ICC of greater than 0.85. The mean scores of each domain of the Thai HAQ and total scores in the second administration were lower than those in the first visit. This finding was not explained by the effects of DMARDs added but might be caused by the advice from the physicians to rest the inflamed joints or the adjustment of nonsteroidal anti-inflammatory drugs and/or analgesics, which should take effect within 1 week.

The method of assessing test-retest reliability of the Thai HAQ by self-administration on the first visit and mailed response 1 week later was similar to those of the Korean and Japanese HAQ studies. Potential biases incurred from a mailed response, such as the incompleteness of filling out the questionnaire and failure to return the response, were avoided.

The Thai HAQ has been demonstrated to correlate moderately with the other measures of RA disease activity. The correlation coefficients between the Thai HAQ and patient global assessment of disease activity and ACR functional class were higher than those between the Thai HAQ and number of tender joints, number of swollen joints, physician global assessment of disease activity, and pain level. Weak correlations were found between the walking domain and number of swollen joints and between the grip domain and number of tender joints, number of swollen joints, and pain level. Newly designed devices (that is, car doors, faucets, and containers) and footwear and improvement of fabric quality (for the wring cloth item) may help improve the patient's ability to perform the activities in the grip and walking domains.

The lowest correlation coefficient was observed between the Thai HAQ and ESR. This finding corroborated with the results from the Arabic, Chinese, and Korean HAQs as ESR correlates better with RA disease duration, radiographic changes, and joint deformity than with functional disability [20]. Three measures of RA disease activity, including patient and physician global assessments of disease activity and ACR functional class, had moderate correlation with all domains of the Thai HAQ. These correlations emphasized the importance of function on the overall health status of patients with RA.

Floor and ceiling effects of the Thai HAQ were not detected at either baseline or month 3 of treatment in this study. These effects were not investigated in the Arabic, Chinese, Japanese, or Korean HAQs. At month 3 of DMARD treatment, very few patients reported the lowest and highest possible scores. Six patients who reported the Thai HAQ score of 0 had significant improvement in their function after DMARD treatment. The only patient who scored 3 was an older patient who already had severe disability at baseline and did not respond to the increment of methotrexate dosage.

Responsiveness of the Thai HAQ measured as SRM was regarded as moderate effect and clinically significant. Large response means (SRM >0.8) were observed for the patient and physician global assessments of disease activity and for pain level. Moderate response means were found for the Thai HAQ (0.75) and number of swollen joints (0.64), whereas the number of tender joints and ACR functional class had the SRM closed to moderate effect (0.59). Our study has shown that patient-reported outcomes, including the Thai HAQ, were more efficient than physician-related outcomes, such as the numbers of tender and swollen joints, in detecting treatment effect. As expected, a small response mean was observed for the ESR (0.41).

## Conclusions

This study, together with our previous work, demonstrates that the Thai HAQ has been shown to be comprehensible, reliable, valid, and sensitive to change in detecting disability in Thai patients with RA. Our findings have confirmed the validity of using the Thai HAQ as an instrument to measure functional status of RA patients after treatment with DMARDs or biologic agents or both in clinical trials and daily practice.

## Abbreviations

ACR: American College of Rheumatology; CI: confidence interval; DMARD: disease-modifying antirheumatic drug; ESR: erythrocyte sedimentation rate; HAQ: Health Assessment Questionnaire; HAQ-DI: Health Assessment Questionnaire Disability Index; ICC: intraclass correlation coefficient; OMERACT: Outcome Measures in Rheumatology Clinical Trials; RA: rheumatoid arthritis; SD: standard deviation; SRM: standardized response mean; Thai HAQ: Thai version of the Health Assessment Questionnaire Disability Index.

## Competing interests

The authors declare that they have no competing interests.

## Authors' contributions

MO initiated the concept and design of the study and collected, analyzed, and interpreted the data and prepared the manuscript and finalized it in accordance with the recommendations. JW, SU, PH, NK, and BS collected the data. All authors read and approved the final manuscript.
